# Diametric effects of autism tendencies and psychosis proneness on attention control irrespective of task demands

**DOI:** 10.1038/s41598-018-26821-7

**Published:** 2018-05-31

**Authors:** Ahmad Abu-Akel, Ian Apperly, Mayra Muller Spaniol, Joy J. Geng, Carmel Mevorach

**Affiliations:** 1 Institute of Psychology, University of Lausanne, Lausanne, 1015 Switzerland; 2 School of Psychology, University of Birmingham, Birmingham, B15 2TT UK; 3 Developmental Disorders Program, Universidade Presbiteriana Mackenzie, São Paulo, 01302-907 Brazil; 4 Department of Psychology, University of California Davis, Davis, California 95616 USA; 5 Center for Mind and Brain, University of California Davis, Davis, California 95616 USA; 6 Center for Human Brain Health, University of Birmingham, Birmingham, B15 2TT UK

## Abstract

Our capacity to attend a target while ignoring irrelevant distraction impacts our ability to successfully interact with our environment. Previous reports have sometimes identified excessive distractor interference in both autism and schizophrenia spectrum disorders and in neurotypical individuals with high subclinical expressions of these conditions. Independent of task, we show that the direction of the effect of autism or psychosis traits on the suppression or rejection of a non-target item is diametrical. In Study 1, in which the presence of a salient non-target item hindered performance, higher autism traits were associated with better performance, while higher psychosis traits were associated with worse performance. In Study 2, in which the presence of a salient non-target item facilitated performance, a complete reversal of effects was observed. Future clinical interventions may be informed by the context-specific advantages we observed for the autism and psychosis spectra, and by the need to consider the diametric effects they yield.

## Introduction

The perceptual saliency of distracting or competing non-target information presents a major challenge for attention selection processes, which are partially driven by the bottom-up salience of objects in the environment^1^. Thus, selection in such a scenario should utilize top-down goal directed processes to bias selection away from distracting, non-target salient items. Independent lines of evidence in autism and schizophrenia spectrum disorder (ASD and SSD, respectively) and the broader spectrum of their traits in neurotypical participants suggest that these conditions are associated with attentional atypicalities under conditions requiring the selection and processing of a target in the presence of a perceptually salient distractor. However, the results in both ASD and SSD have been mixed, with evidence pointing to both impaired as well as intact/enhanced performance. In SSD, the effect of salient distractors on target selection has been associated with both interference^2–5^, and lack of interference, and even benefit (when attention is guided by bottom-up information)^5,6^. Similarly, in ASD, there is evidence for both reduced typical levels of interference^7–9^, as well as increased processing cost^10–13^ in the presence of salient distractors. Thus, the contexts under which ASD and SSD and their expressions in neurotypicals yield beneficial or detrimental effects is currently unclear, not least because it is uncommon for these expressions to be assessed in the same participants.

While ASD and SSD are regarded as distinct conditions, the two conditions exhibit many behavioral, cognitive and neurological similarities^14–17^. In considering their differences, which may be more specific to the psychosis/positive symptom domain, it has been proposed that these conditions are at the opposite extremes of the same cognitive continuum, exerting diametric effects on cognition and behavior^18–22^, putatively due to reciprocal alterations of genetic risk factors^21^. For example, complementary attentional processes may be differentially affected by ASD and SSD, such that individuals with ASD show increased focus of attention and a bias towards local level detail, whilst individuals with SSD (and specifically those with positive symptom schizophrenia) show overswitching and a bias towards global level detail^4,19,23–25^. With respect to distractor suppression, we have shown that autism and psychosis traits, which were assessed in tandem in neurotypical adults, exert mutually opposed effects^18^. Specifically, in this study, we showed that when participants were required to attend to a target (a face) in the presence of salient distractor (a scene) or vice versa, increasing levels of autism traits were associated with reduced interference from the salient distractor, while psychosis traits were associated with increased interference. In a separate study, we also showed that autism and psychosis traits diametrically modulated activity in the anterior portion of the ventral right temporoparietal junction^20^. In this region, which is part of the attention control network, autism traits were associated with increased activity and psychosis traits with decreased activity.

The present study thus has two main aims. First, it examines whether autism and psychosis traits will be beneficial or detrimental depending on the task demand, and (2) whether autism versus psychosis traits would, regardless of the task (context), induce contrasting, diametric effects on target selection in the presence of a non-target, salient distractor. To this end, we conducted two separate studies in neurotypical individuals in whom autism and psychosis traits were assessed in tandem. We test our hypotheses in neurotypical individuals based on the notion that both autism^26^ and psychosis^27^ traits exist on a continuum, ranging from typicality to disorder. This approach has the advantage of eliminating the confounding effects of duration of illness, active symptomatology or medication^28,29^.

In Study 1, participants performed an adapted version of the morphed face-discrimination task^30^. In this task, which required participants to actively ignore/suppress an irrelevant scene, we estimated contrast threshold of the distractor scene at which the participant was still able to correctly identify the face. We predict, similar to our previous results^18^, that, in contrast to psychosis traits, increasing autism traits will be beneficial to performance, i.e., withstanding greater level of distraction while still correctly identifying the face.

In Study 2, participants performed a visual search task^31^ in which a low-contrast target accompanied by a single non-target that was either perceptually similar or more salient had to be identified. Participants knew that the non-target would sometimes be salient (i.e., high contrast) but that the target would never be high contrast. Under this condition, the salient non-target element acts as an anti-cue that directs the participant to the target, and thus facilitates performance^31,32^ (see also^5^). Here, we assessed the benefit gained by the presence of the salient non-target, which is indicative of participants’ ability to reject it and select the target. This benefit was expected as the perceptually salient item was not itself the target, but provided contextual information that could be used to reorient attention toward the target more rapidly^31^. This task was chosen for two reasons: (1) unlike the morphed face-discrimination task, the influence of a salient non-target item in the display would be to improve performance, and (2) there is evidence showing that under conditions similar to the visual search task, higher levels of autism traits were associated with poorer performance and a reduced signal of distractor suppression^13^. If the diametric model is correct, we hypothesize that higher autism traits would be associated with worse performance in this task, whilst higher psychosis traits would be associated with better performance.

To summarize, based on the above discussion, and evidence suggesting that ASD and SSD represent opposite extremes of the same cognitive continuum^18,20,33^, we expected high rates of autism traits to be beneficial in the morphed face-discrimination task (Study 1) but detrimental in the visual search task (Study 2). In contrast, we expected high psychosis traits to be beneficial in the visual search task (Study 2) but detrimental in the morphed face-discrimination task (Study 1). Thus, regardless of the direction of the effect in each task, we predicted opposite effects on performance of the two trait dimensions. If confirmed, in our view, this would constitute the most stringent test, to date, of the diametric model in a neurotypical population^21,34^.

## Results

First, after inspection of the data of the participants from the two tasks, two outliers from the visual search task were excluded from all analyses: one for scoring 5.25 SD above the mean on the positive subscale of the Community Assessment of Psychic Experiences Questionnaire (CAPEp), and one for scoring 5.63 SD below the mean on the saliency benefit score. All other participants fell within 3 SD of the mean. We then examined differences between the two samples in terms of gender distribution, age, Autism Spectrum Quotient (AQ), CAPEp, and Bias, which is the difference between the standardized scores of AQ and CAPEp. The results revealed no significant differences between the samples of Study 1 and Study 2 in terms of gender distribution (χ^2^ = 0.149, p = 0.70), AQ (t_(df=123)_ = 1.38, p = 0.170), or Bias scores (t_(df=123)_ = 1.85, p = 0.067). However, the sample performing the visual search task was older (U = 560.50, Z = 6.87, p < 0.001, η^2^ = 0.38) and had higher CAPEp scores (U = 1198.00, Z = 3.70, p < 0.001, η^2^ = 0.11).

### Performance on the morphed face-discrimination task (Study 1)

First, threshold estimates were significantly higher for male than female participants in the male identification pair condition (M ± SE = 0.70 ± 0.02 *vs* 0.54 ± 0.02; t_(df=56)_ = 4.09, p < 0.001, Hedge’s *g* = 1.29), and marginally so during the female identification pair condition (M ± SE = 0.76 ± 0.02 *vs* 0.71 ± 0.02; t_(df=56)_ = 1.79, p = 0.08, Hedge’s *g* = 0.56). Age was unrelated to threshold estimates for either the male (r_rho_ = 0.19, p = 0.15) or female (r_rho_ = 0.13, p = 0.32) pair conditions. However, threshold estimates were related to familiarity with Robert De Niro (RD; r = 0.43, p = 0.001) and Kevin Spacey (KS; r = 0.27, p = 0.043) in the male condition, and with Margaret Thatcher (MT; r = 0.33, p = 0.013) in the female condition. Moreover, there were no associations between AQ and CAPEp scores with either the familiarity or the identity-classification scores (−0.017 < *r*_*s*_ < 0.11, *p*_*s*_ > 0.43), highlighting that individual differences in autism and psychosis traits did not manifest in overall perceptual performance. Accordingly, threshold scores on the morphed face-discrimination task were adjusted for gender and familiarity ratings. In all regression analyses, the standardized adjusted threshold estimates were entered as the dependent measure and the standardized scores of AQ and CAPEp as the predictors.

**Figure 1:**
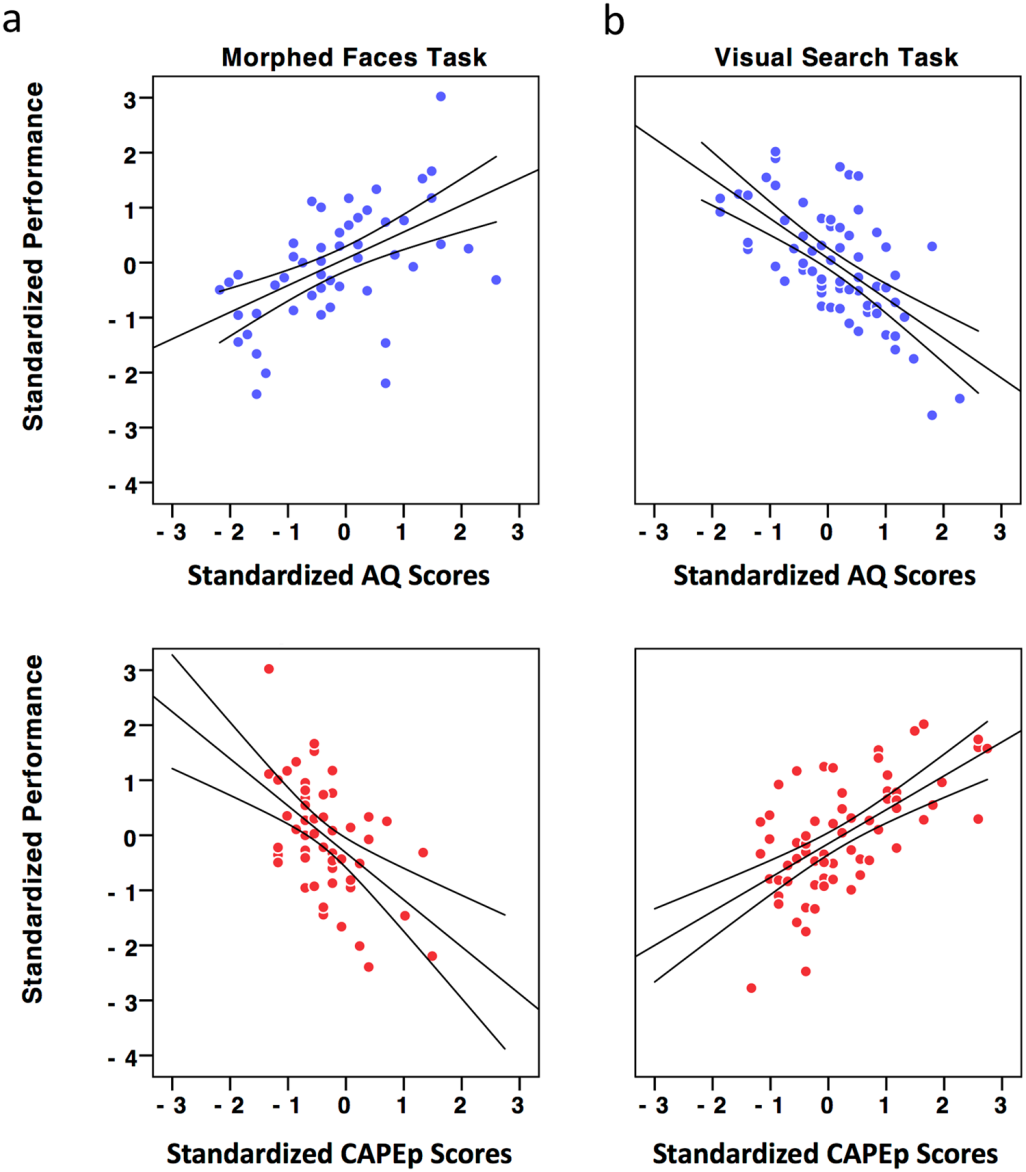
Scatterplot of the standardized performance scores on the morphed face-discrimination of the male pair condition and the visual search task as a function of the standardized autism (AQ) and psychosis (CAPEp) trait scores. Positive values of the standardized performance scores (y-axis) represent better performance. Similarly, positive values on the x-axis represent higher autism/psychosis traits. Panels a and b respectively show the association of autism traits (blue dots) and psychosis traits (red dots) with task performance—On the morphed face-discrimination task (Panel a), higher autism traits are associated with better performance, while higher psychosis traits are associated with worse performance. On the visual search task (Panel b), the reverse pattern of association emerges. All regression lines are significant (p < 0.05).

For the male pair condition, the regression analysis yielded a significant model (F(2,55) = 6.85, p = 0.002, R^2^ = 0.199), explaining approximately 20% of the variance. As can be seen from Fig. 1a, standardized coefficients revealed that while higher autism traits were associated with better performance (β = 0.428, t = 3.18, p = 0.002), higher psychosis traits were associated with worse performance (β = −0.418, t = 3.11, p = 0.003).

For the female pair condition, the model was non-significant (F(2,55) = 0.94, p = 0. 398, R^2^ = 0.033). However, it is noteworthy that, overall, discriminating between the male pairs was significantly more difficult than the female pairs for morphs 3, 4, 6–8 (all *ts*_(df=57)_ > 2.41, all *ps* < 0.019, Hedge’s *g* = 0.10–0.90) (see Fig. S1, Supplementary Information). Moreover, the mean threshold estimate was significantly higher for the female pair (M ± SE = 0.72 ± 0.10) than the male pair (M ± SE = 0.58 ± 0.14) condition (t_(df = 57)_ = 8.76, p < 0.001, Hedge’s *g* = 1.17), suggesting that participants were overall better able to discriminate the female face at higher levels of distraction.

### Performance on the visual search task (Study 2)

In Study 2, the main outcome measure is saliency benefit, which is the benefit gained from the presence of the salient (i.e. higher contrast) non-target compared with the similar contrast non-target. Preliminary analyses (see Fig. S2, Supplementary Information) confirmed the overall benefit gained in the salient versus the similar conditions in terms of accuracy, RT and inverse efficiency (RT/proportion correct). To examine the association of saliency benefit with autism and psychosis traits, we used the inverse efficiency score, which allows us to combine reaction time and accuracy into a single measure^35^.

There was no difference between the saliency benefit scores of the male and female participants (t_(df=65)_ = 0.81, p = 0.419, Hedge’s *g* = 0.23). However, saliency benefit scores were negatively associated with age (r_rho_ = −0.34, p = 0.005). Accordingly, the standardized age-adjusted saliency benefit scores were used as the dependent measure in the regression analysis.

The regression analysis yielded a significant model (F(2,64) = 5.53, p = 0.006, R^2^ = 0.147), explaining approximately 15% of the variance. As can be seen from Fig. 1b, and in contrast to the morphed face-discrimination task, standardized coefficients revealed that while higher autism traits were associated with worse performance (β = −0.310, t = 2.62, p = 0.011), higher psychosis traits were associated with better performance (β = 0.300, t = 2.55, p = 0.013).

### The Bias score

**Figure 2:**
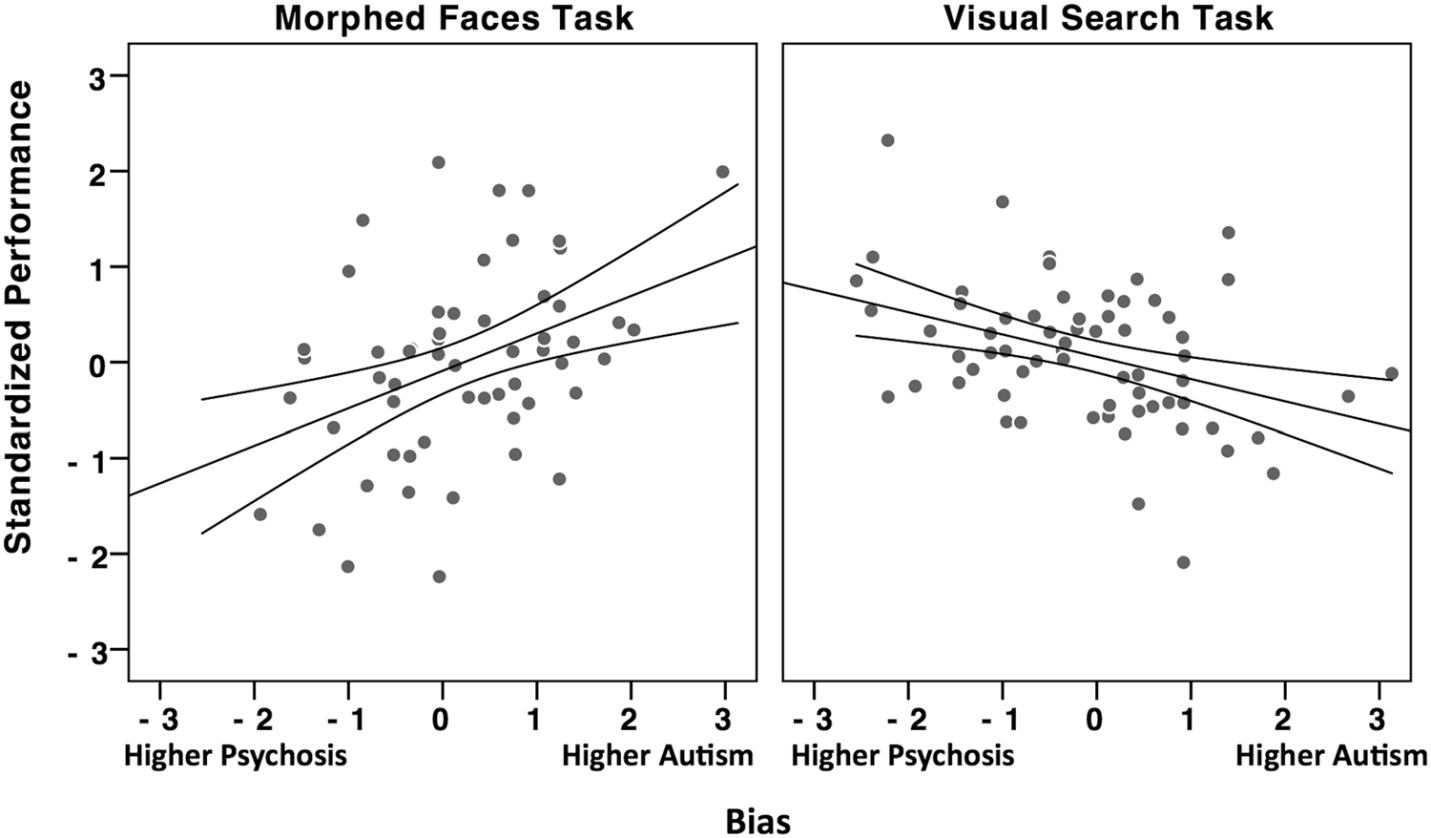
Scatterplot of the standardized performance scores on the morphed face-discrimination and visual search tasks as a function of the relative expression of autism vis-à-vis psychosis scores (Bias = zAQ _minus_ zCAPEp). Negative bias scores indicate dominant expression of psychosis traits (or zCAPEp > zAQ) and positive bias scores indicate dominant expressions of autism traits (or zAQ > zCAPEp). All regression lines are significant (p < 0.05).

In order to examine if performance was associated with the relative dominance of autism versus psychosis traits, we calculated a Bias score for each participant by subtracting their z-normalized CAPEp scores from their z-normalized AQ scores. Regression analyses of both the morphed-faces (F(1,56) = 11.01, p = 0.002, R^2^ = 0.164) and visual search (F(1,65) = 11.13, p = 0.001, R^2^ = 0.147) tasks yielded significant models, explaining 16% and 15% of the variance, respectively. Standardized coefficients confirmed that increasing autism relative to psychosis traits was associated with better performance on the morphed-faces task (β = 0.405, t = 3.32, p = 0.002), and with worse performance on the visual search task (β = −0.382, t = −3.34, p = 0.001) (see Fig. 2).

## Discussion

This study examined the effect of autism and psychosis traits on the processing of a target in the presence of a perceptually competing, non-target element in two scenarios: the morphed-faces task in which a salient distractor incurred processing cost, and the visual search task in which a salient non-target item conferred benefit. As predicted, the effects of autism and psychosis traits depended on the task characteristics. Importantly, however, the results from the two experiments provide converging evidence that autism and psychosis traits have opposing effects on distractor suppression/rejection, and which are not dependent on the specific task demands or contexts. This finding is consistent with a growing body of literature suggesting that ASD and SSD and their extended phenotypic continua within the general population have diametric effects on brain and behavioral phenotypes^4,18–21,36^. It also extends current knowledge by showing that these diametric effects are evident within the attention control system. This is particularly intriguing, as these findings also point to suspected brain mechanisms, specifically in the parietal-occipital and fronto-parietal circuitry (c.f.^31,37^), distinct parts of which may be diametrically modulated by these conditions^20^.

Our results highlight context-specific advantages for autism and psychosis traits. In the morphed face-discrimination task, higher levels of autism traits were beneficial to performance (indicative of improved distractor suppression), while higher levels of psychosis traits were detrimental to performance. These results replicate our previous findings^18^ and further confirm that the effects are unlikely to be attributable to variation in perceptual abilities (which were unrelated to levels of autism or psychosis traits). In contrast, in the visual search task, where the presence of a salient non-target item facilitated performance, higher level of psychosis traits were beneficial to performance (indicative of improvement in the dismissal of the salient non-target item through rapid rejection), while higher levels of autism traits were detrimental to performance. These effects were also discerned as a function of the relative expression of autism vis-à-vis psychosis traits, where increased bias towards autism traits (relative to psychosis) was associated with improved performance on the morphed face-discrimination task, and decreased performance on the visual search task (see Fig. 2). To the extent that the traits measured in our neurotypical participants were on a continuum with clinical ASD and SSD^26,38^, this pattern of results suggests that ASD and SSD have inherent biases in the utilization of attention control.

We suggest that the dual mechanisms theory of proactive and reactive cognitive control^39^ may provide a framework to understand the contrasting effects of autism and psychosis traits we observed in neurotypical individuals, and by extension, the inconsistencies in the literature on attention atypicalities in ASD and SSD^5,6,13,40,41^. In proactive control, individuals bias attention by maintaining goal-relevant information and preventing interference in an anticipatory manner even before the onset of the stimulus. This effect was observed, for example, in Mevorach *et al*.^42^ where participants knew in advance on which perceptual level (global or local) a to-be-ignored element would appear. Contrasting with this, in reactive control, individuals respond “online” to interference after stimulus onset. Under this mode, when a salient non-target is contextually relevant (as in^32^ where it can determine the location of the target), performance may benefit from a process of rapid rejection following stimulus presentation. It is thus possible that dealing with perceptually salient non-target elements in our two tasks preferentially calls upon proactive control (in the morphed face-discrimination task) and reactive control (in the visual search task)—preferentially in the sense that adaptive allocation of attention rarely consists of exclusive reliance on a proactive or reactive mechanism.

With respect to SSD, past research suggests a reliable deficit in proactive control^43,44^, a low dependence on top-down control processes (which relate directly to proactive control)^5^, and no major deficit in the implementation of reactive control^44^. Perhaps the relative reliance of individuals with SSD on reactive control may explain why schizophrenia patients can benefit more than healthy controls from a valid cue (contextually relevant information) and reorient attention toward the target more rapidly^45^. The literature on attention control in ASD is less clear, particularly when previous research has shown that individuals with ASD can be both highly distractible and resistant to distraction^40,41,46^. Intriguingly, however, there is evidence suggesting that top-down attentional control can be typical in ASD when stimuli are static^47^, which may facilitate the use of proactive control. Moreover, similar to our results in the visual search task, individuals with ASD do not benefit from salient non-target elements that facilitate response in typically developing individuals^40^. Notably, the same study^40^ also reported that ASD individuals were less susceptible to distraction from attentional focus, which is consistent with our previous work in neurotypicals showing that the effect of psychosis traits on increasing saliency cost was attenuated in individuals with increased focus of attention as measured by the AQ^18^. However, it is noteworthy that the differences reported in Keehn *et al*.^40^ between ASD and neurotypical individuals may be relative rather than absolute. This is likely, since, as we have shown, attentional control is sensitive to variations of autism traits in neurotypical individuals, and by implications, in individuals with ASD. This underscores the importance of dimensional assessments and individual differences analyses in both clinical and neurotypical populations.

**Figure 3:**
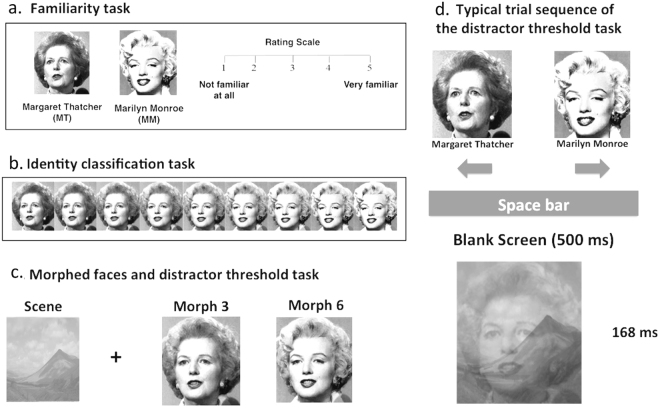
The morphed faces discrimination task. (**a**) Presents the unmorphed faces of the two female celebrities Margaret Thatcher (MT) and Marilyn Monroe (MM), which were judged on familiarity from 1 (unfamiliar) to 5 (very familiar). (**b**) Presents the continuum of morphed faces from MT to MM in 10% increments. (**c**) Presents the distractor scene and the morphed female faces used in the distractor threshold task. (**d**) Presents a typical trial sequence in the distractor threshold task. The combined stimulus is of MT’s face at 20% contrast and the scene at 50% contrast. The scene is from https://www.flickr.com/photos/aaranged/8804736077/in/photolist-eq. 3zhr-Wxp3fR-cQn49h-8CfRWR-6Y3wHR-7JkauQ-rQ7UoK-c79nqG-c79ydJ-6Y3KTE-nvSYcK-Cn2d2C-c784Fy-Fqd8Pp-6XYFeF-cQnoFY-HsBfRJ-rkm6oy-8CbMES-7CHU2i-51rzKT-UvvP4b-55etfL-7wkbH5-Zqk4HW-W3eg4a-ZoUKYq-afKcrw-cSaHaL-64rsnE-XLJkRv-ZoUHpN-8yxzGP-aztt3V-7Lndd7-Cn1b9w-8tnrXv-pu4Frd-cQmUq9-53MXCU-Cn22PU-73fsHG-591tQ-DhLfd3-j4nkVJ-83MBDd-7arEtU-FPDpEX-9qE7yV-oEPvEH (Creator, Aron Bradley; License, https://creativecommons.org/licenses/by-sa/2.0/). The images of MT and MM were adapted with permission from Springer Nature [Rotshtein, P., *et al*. 2005. Morphing Marilyn into Maggie dissociates physical and identity face representations in the brain. *Nature Neuroscience* 8, 107–113]. Note: This scene is for illustrative purposes, as the original scene used in the current study could not be displayed due to copyright restrictions. Male celebrity images could not be displayed due to copyright restrictions. We highlight that data were collected between 2015–2016.

Our results may also be considered in terms of the attentional tradeoff model of Amer *et al*.^48^. According to this model, task performance depends on cognitive control mechanisms associated with diffused and focused attention, which respectively facilitate the use and suppression of irrelevant/distracting information. These two components appear to map onto the attentional profiles of ASD and SSD, which, as pointed out in the introduction, are respectively associated with tendencies for the deployment of focused and diffused attention^4,19,23–25^. However, while proactive and reactive control refers to control aspects and temporal dynamics of top-down processes regardless of the perceptual process at hand, diffused and focused modes may also reflect or bias specific perceptual processes. For instance, diffused attention may facilitate global or holistic processing while focused attention may facilitate local or part-based processing. While these fit with some of the previous findings in the ASD and SSD literature^3,4,8^, we argue that they are less likely to explain our findings. As we have shown here, individual differences have not been associated with perceptual performance in the identity-classification task (Fig. 3b), where perceptual processes would have likely affected performance. Furthermore, diffused processing should have benefited overall performance in the visual search task, including in the similar trials. Accordingly, one would expect psychosis traits to confer benefit in the similar trials as well. However, we found no association between psychosis traits and the participants’ accuracy, reaction times, or efficiency scores on the similar trials (−0.10 < *rs* < 0.04, *ps* > 0.40). This is consistent with evidence showing that performance of schizophrenia patients suffers in visuospatial tasks that require broad monitoring^49^. Furthermore, even when explicit global/local identification was tested^18^, the diametric effects, we reported, held irrespective of the level of processing. This highlights, once more, that the association of autism/psychosis traits with the attention control mechanisms, we assess here, transcends perceptual biases.

The notion of overselectivity in ASD^50^ and its interaction with proactive vs. reactive control can also be considered here. Although not a defining behavior in ASD, overselectivity refers to the tendency to select only a subset of the input. Critically, however, this again implies difficulty in more holistic or global processing and therefore suffers from a similar caveat in its ability to explain our present findings. Nevertheless, to the extent that overselectivity can be adapted according to task demands (so that different aspects of the input could be highlighted for selection, including global over local aspects), it can provide a plausible framework for understanding improved proactive distractor inhibition in ASD. Specifically, we can conceptualize that setting up the ‘overselective’ attention mechanism in preparation (before stimulus presentation) is intact in ASD and, therefore, conveys performance benefit due to superior selectivity (i.e., distractor filtering). However, adapting the selective mechanism ‘on the fly’ based on the current stimulus may be more problematic. Thus, the former would be a manifestation of proactive control, while the latter would be a manifestation of reactive control. This may also highlight that overselectivity per se is not necessarily an impediment to behavior. Problems occur only if it cannot be adapted (which is perhaps where remediation attempt should focus). Taken together, our results provide important insight into the mechanism of attentional control and its susceptibility to attenuated subclinical expressions of ASD and SSD.

We are aware of the debate regarding the extent to which non-pathological individuals with no pathological symptoms constitute an appropriate model for pathologic functioning. With this in mind, and to the extent that a dimensional view of psychopathology is accepted^26,27,51^, our findings can have important implications for interventions within the clinical populations, as they identify benefits for ASD and SSD that depend on the mode of control (possibly reactive versus proactive) that is necessary in a given context. In this regard, we propose that the interference of irrelevant salient elements can be mitigated in SSD in contexts that preferentially require reactive mode of attentional control, and in ASD in contexts that preferentially require proactive mode of attentional control. More generally, such diametric associations suggest that ASD and SSD are affected by reciprocal causes, which, in turn, can be developed into reciprocal treatments. Indeed, it has been pointed out that contrasting pharmacological agents, such as antagonists and agonists of metabotropic glutamate receptor 5 (mGlur5) pathways, are being developed for the treatment of ASD and SSD, respectively^22^. An immediate step of practical significance, however, would be to routinely assess both conditions in both populations, particularly when such trait combination (or the relative symptom expression of one condition vis-à-vis the other) appears to determine clinical functional outcomes^52^.

Our results need to be interpreted in light of some limitations. It is notable that one key difference between the visual search and morphed-face discrimination tasks employed here is that they rely on different selection ‘units’. The morphed-face discrimination task (Study 1) incorporates object-based selection, while the visual search task (Study 2) incorporates both feature- and space-based selection. Could it be that autism traits are associated with improved object-based attention while psychosis traits are associated with improved space-based selection? We argue that this is unlikely, particularly in light of robust evidence for superior performance of individuals with ASD in visuospatial search tasks^4,53^. Another alternative explanation for the benefit of high autism traits in the morphed face-discrimination task may relate to the putative bias in ASD for local processing^4,54^. However, this again is unlikely to be the case here. The local bias is typically associated with poor face identification^55^, and previous work has shown less interference of salient distractors in individuals with high expressions of autism traits across both faces and global-local tasks^18^. In addition, our identity-classification task (Fig. 3b) was used exactly in order to exclude such potential association with perceptual abilities. Indeed, autism traits were unrelated to performance on the identity-classification task when distractors were not presented (and therefore not suppressed) (see also^56^ for a similar approach). Taken together, these findings suggest that the better performance of individuals with high autism traits in the morphed face-discrimination task is unlikely to be due to some specific autism-related proclivity for face identification, emphasizing the notion that only when attention control was called upon that differences emerged.

Moreover, although no gender effect was observed, some caution regarding the generalizability of our findings is warranted, given that our sample consisted largely of female participants. This might be of concern regarding the effect of autism traits where the male-to-female ratio is about 3:1. However, the fact that effects were discerned in female-dominant samples might attest to their robustness. It is thus reasonable to predict stronger effects still in samples in which males are more represented. Finally, in the morphed face-discrimination task, we obtained significant effects only in the male pair condition. Because of the high level of performance in the female relative to the male pair condition, the absence of these effects in the female pair condition might be explained by a potential ‘ceiling effect’. We thus do not consider this a ‘failure to replicate’ per se, but rather a case where the female pair condition was not sufficiently sensitive.

It is difficult to draw from previous literature clear-cut dichotomies on how ASD and SSD activate attentional control. However, our results confirm that ASD and SSD expressions have diametric effects on distractor suppression, and dependent on the context in which the stimulus is presented, the expressions of these conditions can be associated with performance advantages. Moreover, as can be inferred from the bias score analysis (Fig. 2), attentional control in ASD and SSD might be better explained by considering the relative, rather than the absolute, expression of the symptoms of these conditions within the individual. The latter point behooves a shift in both research and clinical practice where ASD and SSD symptom expressions ought to be assessed simultaneously, particularly in light of the unique diametric effects these conditions appear to have on brain and behavioral outcome measures. This would be an important step forward if we were serious about the need to build multidimensional models of psychopathology.

## Method

### Participants

The two experiments were conducted in two independent samples of healthy adults. In Study 1, 58 participants completed an adapted version of the morphed face-discrimination task^30^, and in Study 2, 69 participants completed the visual search task^31^. The sample size of Study 1 was based on studies examining differences between neurotypical groups with low and high autism/psychosis traits^4,57^, and the sample size of Study 2, was based on Study 1. All participants had normal or corrected-to-normal vision, and self-reported that they had no history of psychiatric illness, epilepsy, neurological disorders, or brain injury, current or past alcohol and/or substance abuse problems. The University of Birmingham Research Ethics Committee approved the study, and written informed consent was obtained from all participants.

Autism and positive psychosis traits were respectively assessed with the Autism Spectrum Quotient (AQ)^26^ and the positive subscale of the Community Assessment of Psychic Experiences Questionnaire (CAPEp)^38^. The AQ is a self-report questionnaire and consists of 50 items that measure the presence of traits associated with the autistic spectrum within the general population. It consists of five subscales, with moderate to satisfactory reliabilities (Cronbach’s α = 0.63–0.77)^26^, that assess communication, social skills, attention to details, attention switching/strong focus of attention and imagination. Responses are on a four-point scale from “Definitely Agree” to “Definitely Disagree”, and each item is given a score of 0 or 1. AQ’s overall scores can range from 0–50. The CAPE positive subscale consists of 20 items, and has satisfactory reliabilities (Cronbach’s α = 0.82–0.85)^36,58^. Items are scored on a Likert frequency scale from 1 (never) to 4 (nearly always), and scores can range from 20–80. The internal consistency of both scales and for both samples is reasonable (Cronbach’s α = 0.73–0.82). Demographics and characteristics of participants in Study 1 and Study 2 are presented in Table 1.

**Table 1:** Sample demographics and characteristics in Study 1 and Study 2.

Face-Discrimination Task (Study 1) (N = 58)				Visual Search Task (Study 2) (N = 69)			
Gender (MF)	Age^a^ M (SD)	AQ M (SD)	CAPEp M (SD)	Gender (MF)	Age^b^ M (SD)	AQ M (SD)	CAPEp M (SD)
13/45	20.95 (3.80)	16.78 (7.02)	26.10 (3.83)	19/50	26.26 (4.06)	18.45 (5.51)	30.49 (7.37)

^a^Age range: 18–34 years

^b^Age range: 17–36 years; AQ = Autism Quotient; CAPEp = Positive scale of the Community Assessment of Psychic Experiences questionnaire; MF = Male/Female; M (SD) = Mean and Standard Deviation.

### Study 1: The morphed face-discrimination task

In Study 1, we used an adapted version of the morphed face-discrimination task^30^ superimposed with a variable contrast scene distractor (see Fig. 3). The experiment consisted of three stages. In the first (face familiarity task), the purpose was to evaluate how familiar participants were with two pairs of famous faces—a female pair (Margaret Thatcher and Marilyn Monroe; see Fig. 3) and a male pair (Robert De Niro and Kevin Spacey; see note in legend of Fig. 3). Faces are presented on a grey background. Participants had to rate how well they knew each celebrity, on a Likert scale from 1 to 5, with 1 being ‘not familiar at all’, and 5 being ‘very familiar’, that is, knowing who the celebrity was and even knowing facts about the celebrity (see Fig. 3a). In the second stage (the identity-classification task), the famous male and female faces were paired by gender and morphed together. The faces were morphed in varying degrees of identity change, starting from 10% of face 1 (e.g., Margaret Thatcher), going through nine different morph levels in steps of 10% and finishing at 90% of face 2 (i.e., Marilyn Monroe) (see Fig. 3b). The identity-classification task for each of the pairs started with the unmorphed faces and their names, presented on the screen side by side. The morph was then presented for 750 ms followed by a fixation point that remained on the screen until a response was made. Response time was not measured. Participants responded by pressing the left or right key to indicate which of the previously presented unmorphed faces was more like the morphed face presented in each trial. The identity-classification for each morph continuum consisted of 27 trials presented in random order (3 presentations for each morph).

As we were interested in unearthing individual differences, we selected 2 points from the morphed continuum that yielded the greatest performance variability in a pilot run of the classification task with a number of different face pairs to be used in the final stage of the experiment. Consequently, in the third stage (distractor threshold task), morphed faces 3 and 6 from each continuum (Fig. 3c) were combined with an irrelevant scene that was superimposed on top of the morphed faces. Participants were asked to ignore the scene and identify the faces. As in the previous task, the unmorphed female and male faces were first presented on the screen, before the start of the run. After pressing the space bar, a blank screen appeared for 500 ms. Participants were then presented with one of the morphed faces (at 20% contrast) superimposed with the scene for 168 ms (see Fig. 3c,d). Responses were recorded (with no time constraint) by pressing the left or right key to indicate if the face corresponded to the initially presented exemplar on the left or right side of the screen. The contrast of the scene would vary according to an adaptive staircase procedure, with a 3-down-1-up structure, which changed the contrast by 10% in each step. In each block, two interleaved staircases were run, one with a high contrast starting value (50%) and another with a low contrast starting value (25%). Each staircase was dropped after 7 reversals, resulting in a varied number of trials per participant per block. The task consisted of 6 blocks (3 for the male and 3 for the female faces). The threshold estimate for each staircase was extracted as the average of the reversals. The overall contrast threshold was calculated as the average of the 6 staircase procedures (2 per block). The contrast threshold ranges from 0–1, and is a measure of the participant’s ability to identify the correct face with the maximum contrast possible from the distracting scene at 72% accuracy. Thus, efficient distractor suppression in this task results in higher thresholds.

This task constitutes a replication test of our previous study using the face-scene task^18^, but with the additional improvement of controlling for the potential effect of perceptual abilities on distractor suppression, which we accounted for here in step ‘a’ and ‘b’ of the task (see Fig. 3a,b). It is also worth noting that the design of this task, with prior knowledge about the to-be-ignored distractor (i.e., the scene which is always the same) and the location certainty, mimics previous investigations highlighting the role of the intraparietal sulcus (IPS) in suppression of salient distractors^59,60^. Here, calling upon attentional control in advance of stimulus presentation can mitigate the interference from the scene and thus can increase the participants’ distractor threshold. Conversely, impaired suppression (e.g., following TMS to the IPS) has been previously shown to reduce the amount of distractor/noise signal that can be effectively filtered out in such paradigms^59,60^.

The experiment was programmed and administered using PsychoPy^61^. Participants sat 60 cm from a 17″ Samsung SyncMaster 720 N LCD monitor, set to the native resolution of 1280 × 1024 at 75 Hz. All faces and distractor stimuli were presented centrally on this monitor and measured 6 cm width × 6.5 cm height (subtending 5.75° of visual angle horizontally and 6.18° of visual angle vertically).

### Study 2: The visual-search task

In the visual search task^31^, an occasional salient non-target element acts as an anti-cue, redirecting attention towards the target. In this task, participants searched for a low-contrast target that co-appeared with a single non-target that was either perceptually similar (i.e., similar contrast) or salient (i.e., higher contrast). Participants were informed in advance that the non-target would sometimes be high contrast but that the target would never be high contrast. Previous work has demonstrated that even when subjects attend or saccade to the salient distractor, knowledge of the anti-correlation between the salient stimulus and the target can be used to improve performance compared to the similar condition^32,62^. This presumably occurs because the salient distractor is attended, but can easily be reactively suppressed, releasing attention to select the actual target. In contrast, when the similar distractor is erroneously attended, it requires a greater degree of scrutiny in order to reject as a non-target.

**Figure 4:**
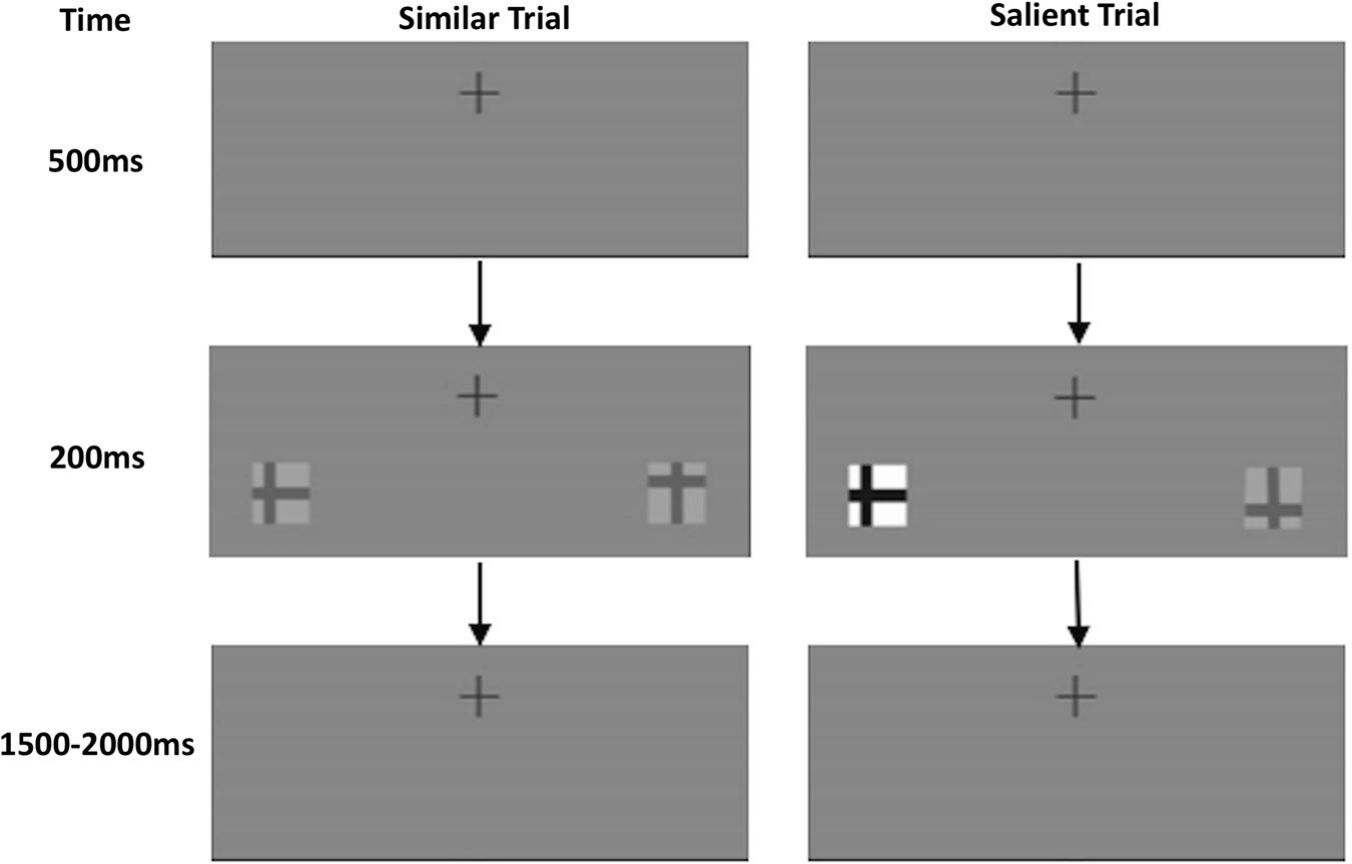
Trial procedure of the visual search task in the similar and salient conditions. A trial begins with a fixation cross that appears for 500 ms. This is followed by a 200 ms search display. On 50% of the trials, the target co-appears with a similar contrast stimulus. On the other 50% of the trials, the target co-appears with a salient (i.e., higher contrast) one. The trials were separated by a variable fixation interval ranging from 1500 to 2000 ms.

In this task, participants were presented with two ‘*t*’-like stimuli for 200 ms (see Fig. 4, and^31^ for full details). The non-target *t* was rotated 90° to the left or right from vertical, and the target *t* was either upright or inverted. The target and non-target stimuli appeared randomly in the left and right visual fields (one on each side). Participants were asked to find the target and indicate whether it was upright or inverted using manual key presses. In 50% of the trials, the non-target was similar in contrast to the target and thus non-salient (Michelson contrast ratio = 0.45; foreground luminance = 35.5 cd/m^2^; background luminance = 93.5 cd/m^2^), and in 50% of the trials the non-target had higher contrast (Michelson Contrast Ratio = 0.91; foreground luminance = 7.1 cd/m^2^, background luminance = 160.3 cd/m^2^) and thus more salient. The horizontal distance of the closest part of the stimuli to fixation was ±2.95° of visual angle, and the vertical distance was −0.85° of visual angle. The stimuli subtended 0.85° visual angle at fixation. The target was equally likely to appear in the left and right visual fields. The background throughout the experiment was an intermediate gray (77.8 cd/m2, as in^31^).

The trials were separated by a variable fixation interval ranging from 1500 to 2000 ms. This feature makes temporal expectancies harder and thus induces more attentional control post-stimulus presentation. The outcome measure of interest in this task is the saliency benefit, which is the difference between the performance in the salient and similar conditions (i.e., Similar_minus_ Salient conditions).

### Statistics

We performed parametric (Pearson, t-test, ANOVA, regression) and non-parametric tests (Spearman’s rho, Mann-Whiteny U, Chi-Square), as appropriate. Parametric tests were applied to all dependent variables observing or approximating normality. We applied non-parametric tests to examine differences in gender distribution and in CAPEp scores, which were positively skewed. For the main analyses, in which we were interested in examining the effect of task on performance in individuals with various levels of autism/psychosis traits, we performed individual differences analysis using linear regressions. In addition, individual differences analyses were also conducted by examining the association of the relative expression of autism vis-à-vis psychosis traits with performance on each task. This is based on previous research showing that performance on social and attentional tasks and associated brain activity is associated with the relative dominance (or Bias) of one trait dimension over the other^18,20,36^. For all regression analyses, we report the standardized beta coefficients. All tests applied here were two-tailed tests, and effect sizes were computed using η_p_^2^, η^2^, R^2^, or Hedge’s g, as appropriate.

### Data Availability

The datasets generated during and/or analyzed during the current study are available from the corresponding authors on reasonable request.

## Electronic supplementary material

Supplementary Information
