# Supplementary material for: Diametric effects of autism tendencies and psychosis proneness on attention control irrespective of task demands
Source: Sci Rep. 2018 May 31;8:8478. doi: 10.1038/s41598-018-26821-7 (PMC5981437; doi:10.1038/s41598-018-26821-7)
Supplement: Supplementary file 1 — Supplementary Information [file 41598_2018_26821_MOESM1_ESM.pdf]

Title: Diametric effects of autism tendencies and psychosis proneness on attention control irrespective of task demands

Authors: Ahmad Abu-Akel[1*], Ian Apperly[2], Mayra Muller Spaniol[3], Joy J. Geng[4,5], Carmel Mevorach[2,6*]

Affiliations:

[1]Institute of Psychology, University of Lausanne, Lausanne 1015, Switzerland

[2]School of Psychology, University of Birmingham, Birmingham B15 2TT, UK

[3]Developmental Disorders Program, Universidade Presbiteriana Mackenzie, São Paulo 01302-907, Brazil

[4]Department of Psychology, University of California Davis, Davis, California 95616, USA

[5]Center for Mind and Brain, University of California Davis, Davis, California 95616, USA

[6]Center for Human Brain Health, University of Birmingham, Birmingham B15 2TT, UK

* Correspondence and requests for materials should be addressed to: A.A. (email: ahmad.abuakel@unil.ch) or to C.M. (email: C.Mevorach@bham.ac.uk).

Supplementary Information

Performance on the morphed face-discrimination task (Study 1)

Figure 1S presents the participants' performance on the morphed face-discrimination task during the male and female pair conditions.

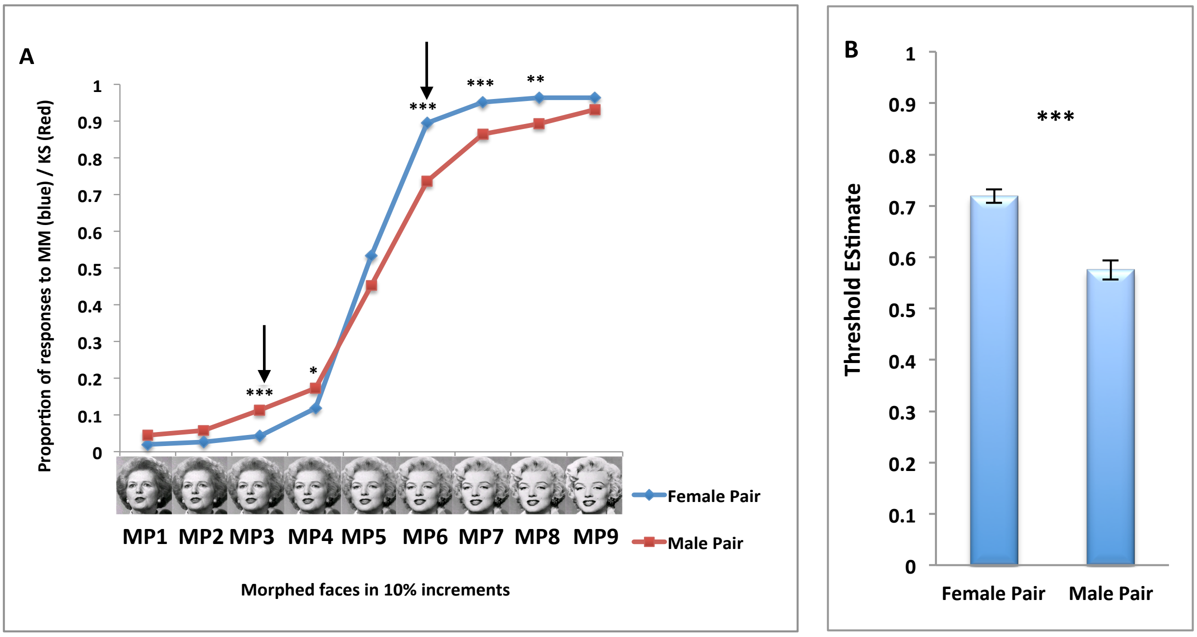

Figure 1S. Performance on the morphed faces-discrimination task during the male and female pair conditions. **Panel A** shows the results of the identity-classification task at all morph levels of the male and female pair condition. Comparisons between the two conditions revealed significant differences for morphs 3, 4, 6-8 (all $ts_{(df=57)} > 2.41$, all $ps < 0.019$, Hedge's $g = 0.10$—0.90). **Panel B** shows the results from the face-scene distractor threshold task during the female and male pair conditions. Mean threshold estimates are higher in the female versus the male pair condition ($t_{(df=57)} = 8.76$, $p < 0.001$, Hedge's $g = 1.18$). The pattern of results suggests that discriminating between the male pair was significantly more difficult than the female pair. Black arrows indicate the set of pairs used in the face-scene distractor threshold task. Error bars represent SEM. * $p < 0.05$; ** $p < 0.01$; *** $p < 0.001$.

Note: The images of MT and MM were adapted with permission from Springer Nature [Rotshtein, P., et al. 2005. Morphing Marilyn into Maggie dissociates physical and identity face representations in the brain. *Nature Neuroscience* 8, 107-113]. Morphing sequence for the male pair could not be displayed due to copyright restrictions.

Performance on the visual search task (Study 2)

The main outcome measure in this task is saliency benefit, that is, the benefit gained from the presence of the salient (i.e. higher contrast) non-target compared with the similar non-target. To ascertain the benefit gained in the salient versus the similar condition, we conducted a series of repeated measures analyses of variance for the overall sample (N=67) in terms of accuracy, reaction time (RT), as well as in terms of RT/proportion correct. The analyses show that, overall, particpants are more accurate ($F(1, 67) = 11.10$, $p = 0.001$, $\eta_p^2 = 0.144$; Figure 2SA), faster ($F(1, 67) = 22.38$, $p < 0.001$, $\eta_p^2 = 0.254$; Figure 2SB), and have lower RT/proportion correct scores ($F(1, 67) = 27.61$, $p < 0.001$, $\eta_p^2 = .295$; Figure 2SC) in the salient versus the similar condition. These findings confirm the benefit rendered by the presence of the salient non-target element.

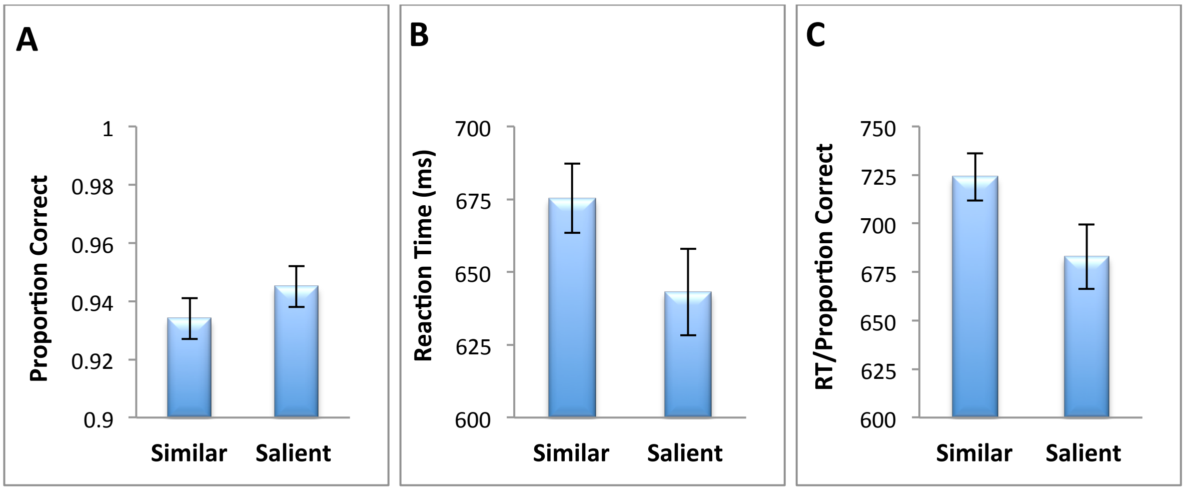

Figure 2S. Overall performance in terms of accuracy, reaction time and reaction time/proportion correct on the similar versus the salient conditions of the visual search task. **Panel A** shows the performance in terms of accuracy. **Panel B** shows the performance in terms of reaction time. **Panel C** shows the performance in terms of reaction time/proportion correct. Bars represent SEM.
